# Grice Arthrodesis and Arthroereisis in the Surgical Management of Pediatric Flatfoot: Radiological Outcomes and Limitations

**DOI:** 10.3390/jcm15020509

**Published:** 2026-01-08

**Authors:** Harun Marie, Alexandru Herdea, Ruxandra Ilinca Marica, Alexandru Ulici

**Affiliations:** 1 11th Department of Pediatric Orthopedics, Carol Davila University of Medicine and Pharmacy, Bulevardul Eroii Sanitari Numarul 8, 050474 Bucharest, Romania; alexandru.ulici@umfcd.ro; 2 Pediatric Orthopedics Department, Grigore Alexandrescu Children’s Emergency Hospital, 011743 Bucharest, Romania; harun_marie@spitalulgrigorealexandrescu.ro (H.M.); ilinca.marica@stud.umfcd.ro (R.I.M.)

**Keywords:** Achilles lengthening, arthroereisis, foot deformities, foot surgery, Grice arthrodesis, pediatric flatfoot, pes planus, radiographic outcomes, subtalar joint

## Abstract

**Background:** Flexible flatfoot is a common pediatric condition. Surgical intervention is indicated for symptomatic cases unresponsive to conservative treatment. This study evaluates the outcomes of two established procedures, Grice extraarticular subtalar arthrodesis and subtalar arthroereisis, in children treated for symptomatic flatfoot. **Methods:** A retrospective analysis was conducted on 158 patients (286 feet) treated between 2013 and 2024. Among them, 34 underwent Grice arthrodesis and 124 underwent arthroereisis. Demographic and procedural data were collected, including age, sex, neurological impairment (cerebral palsy), laterality, and concurrent Achilles tendon lengthening. Radiographic parameters assessed pre- and postoperatively included Meary’s, Pitch, and Kite’s angles (frontal and sagittal view), uncovering of the talus, and Cyma line. Only patients with both pre- and postoperative measurements were included in paired analysis. Statistical tests included paired *t*-tests within groups and Welch’s *t*-tests for between-group comparisons. **Results:** Grice patients were younger (mean age 9.0 ± 3.1 years) and included all cerebral palsy cases (18/34; 52.9%), while arthroereisis patients were older (10.8 ± 2.6 years) and typically neurologically normal. Achilles tendon lengthening was performed in 100% of Grice and 48% of arthroereisis cases. Both groups showed significant radiographic improvement across all measured parameters (all *p* < 0.05). Grice arthrodesis produced greater reductions in Meary’s angle (right Δ = −19.8° ± 9.2 vs. −13.1° ± 7.5; *p* = 0.024), while arthroereisis yielded larger increases in Pitch angle (left Δ = +9.2° ± 7.2 vs. +5.5° ± 6.2; *p* = 0.055). Other angular improvements (Kite’s, uncovering, and Cyma line) were statistically significant within both groups but not between groups. **Conclusions:** Symptomatic flat-valgus foot in children remains a relevant public health issue. Treatment should be individualized, while cases secondary to unrecognized or untreated congenital conditions often require surgery to restore normal foot biomechanics.

## 1. Introduction

Flatfoot (pes planovalgus) represents one of the most frequent deformities encountered in pediatric orthopedics, characterized by a decrease or collapse of the medial longitudinal arch, valgus deviation of the hindfoot, and forefoot abduction [1,2]. While physiologic flatfoot is common in toddlers and tends to resolve spontaneously as the musculoskeletal system matures, a subset of patients remains symptomatic, presenting with pain, fatigue during ambulation, or functional impairment that warrants medical or surgical intervention [3,4].

The etiology of pediatric flatfoot is multifactorial, involving ligamentous laxity, obesity, neuromuscular disorders, or structural abnormalities such as tarsal coalitions [5,6]. The condition is generally classified as flexible or rigid, with the flexible variant being far more prevalent [7]. In most cases, conservative management including orthotic support, stretching exercises, and physical therapy constitutes the first line of treatment; however, persistent symptomatic deformities unresponsive to conservative care may require surgery [8].

Historical perspectives on pes planus have changed significantly over time. As summarized by a review in 2023, flatfoot was traditionally viewed as a pathologic deformity associated with weakness and poor physical function, which led to aggressive use of orthoses and early corrective interventions. Modern biomechanical and developmental research, however, demonstrates that most flexible flatfeet in children are physiological variants, with symptoms, other than arch height determining the need for treatment. This shift from a purely morphological to a functional understanding of flatfoot underpins contemporary interest in procedures that restore alignment while preserving subtalar mobility, such as arthroereisis, and contrasts them with more stabilizing options like the Grice arthrodesis [9].

Flexible flatfoot tends to resemble a normal foot after age 5–6 and remains flexible at any age, whereas rigid flatfeet caused by congenital malformations (vertical or oblique talus) do not correct spontaneously and require early diagnosis and treatment [10]. Multiple surgical options have been described for correcting symptomatic flatfoot, including extra-articular subtalar arthrodesis (Grice procedure), subtalar arthroereisis, and calcaneal osteotomies [11,12]. The Grice arthrodesis, first described in 1952 for paralytic valgus deformities, involves placing a cortical bone graft between the talus and calcaneus to block excessive eversion while maintaining growth potential [13]. In contrast, subtalar arthroereisis is a minimally invasive technique in which a small implant is inserted into the sinus tarsi to limit pronation and restore the physiological arch [1,14].

Both procedures have demonstrated favorable clinical and radiological outcomes in children [13,15,16], yet they differ in invasiveness, rehabilitation time, and long-term stability. The Grice technique remains preferred for younger patients and those with neurological impairment, whereas arthroereisis is favored in older children with flexible deformities and preserved muscle balance [17,18].

Despite the widespread use of both procedures, there is no consensus regarding their optimal indications, comparative radiographic effectiveness, or age-related biomechanical advantages. Most available studies evaluate each technique separately, often in small cohorts, and direct comparative data remain scarce, particularly in pediatric populations with mixed etiologies. Furthermore, surgeons frequently face overlapping indications—older children with flexible idiopathic deformities may be candidates for both procedures, while younger patients with mild neuromuscular imbalance represent a gray zone in which evidence-based guidelines are lacking. This absence of objective comparative analysis has created significant variability in surgical decision-making, underscoring the need for studies directly evaluating radiographic outcomes between Grice arthrodesis and arthroereisis. The present work aims to address this gap by providing a large-scale, radiographically focused comparison of both techniques within a single institution over more than a decade.

Given the ongoing debate regarding the optimal surgical approach, this study aims to analyze and compare the indications, limitations, and radiographic outcomes of the Grice extra-articular subtalar arthrodesis and the subtalar arthroereisis in the treatment of pediatric flatfoot. By correlating clinical and imaging findings, the present work seeks to define evidence-based criteria that may guide surgical decision-making and optimize long-term functional results in this population.

## 2. Materials and Methods

### 2.1. Study Design and Patient Selection

This was a single-center, retrospective comparative cohort study of pediatric patients (<18 years) who underwent surgical correction for symptomatic flexible flatfoot using either Grice extra-articular subtalar arthrodesis or subtalar arthroereisis between January 2015 and December 2024.

Inclusion criteria were patients younger than 18 years with symptomatic flexible flatfoot unresponsive to conservative treatment, with complete preoperative and postoperative clinical and radiographic data, and a minimum follow-up of 12 months. Exclusion criteria included patients with tarsal coalitions, previous foot surgery, post-traumatic deformities, incomplete clinical or radiographic data, or less than 12 months of postoperative follow-up. Clinical, demographic, and radiographic parameters were obtained from electronic medical records and operative notes.

Because this is a retrospective study, the choice between Grice extra-articular arthrodesis and subtalar arthroereisis reflected the surgeon’s choice. Grice arthrodesis was preferentially selected for younger patients, typically under 9–10 years of age, and for those with neuromuscular disorders. In a growing child, the gradual resorption of the autologous bone graft over time indicates maintenance of the achieved correction, while allowing restoration of physiological mobility at the level of the sinus tarsi.

In contrast, subtalar arthroereisis was applied in older children and adolescents with flexible idiopathic flatfoot. Achilles tendon tightness influenced the selection only secondarily; equinus was addressed surgically when present but did not dictate the primary procedure. All decisions were made by senior pediatric orthopedic surgeons with experience in pediatric foot and ankle deformity correction.

### 2.2. Surgical Techniques

**Figure 1:**
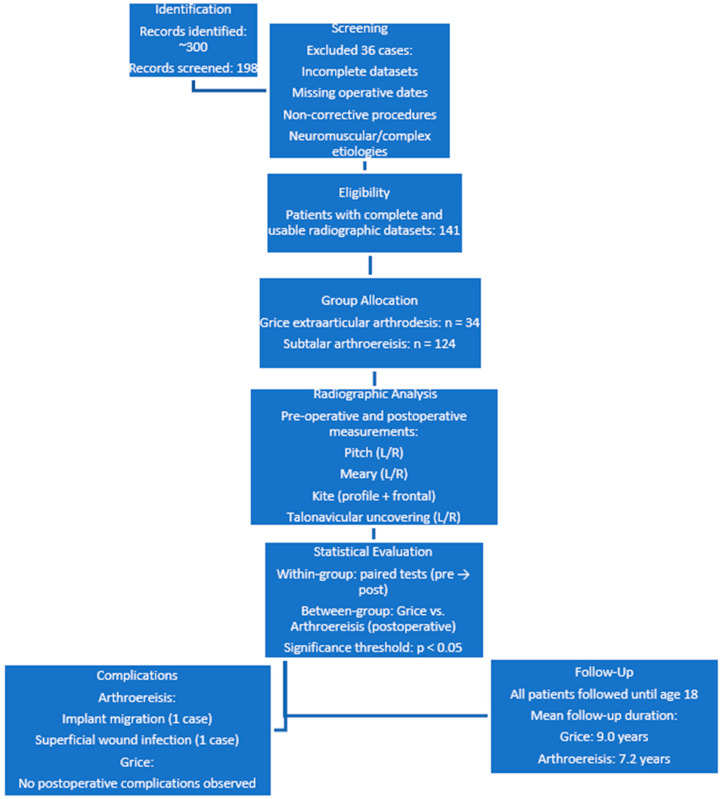
Flow diagram illustrating patient identification, screening, eligibility assessment, group allocation, radiographic analysis, statistical evaluation, complications, and follow-up in a retrospective cohort of pediatric patients treated surgically for symptomatic flexible flatfoot. The diagram details reasons for exclusion, the final number of patients with complete radiographic datasets, allocation to Grice extra-articular subtalar arthrodesis or subtalar arthroereisis, radiographic parameters analyzed, and follow-up duration.

The Grice procedure was performed through a lateral approach to the subtalar joint. After preparation of the sinus tarsi, peroneus graft was inserted to achieve extraarticular subtalar arthrodesis while preserving articular surfaces, as seen in Figure 1. In all Grice cases, Achilles tendon lengthening was performed using a vulpius technique when equinus deformity was identified.

**Figure 2:**
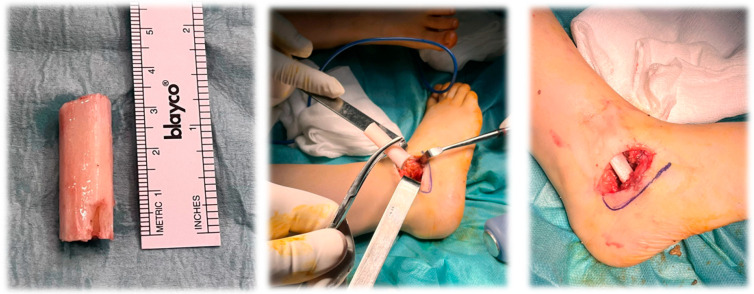
Intraoperative images of Grice extraarticular subtalar arthrodesis: the first image shows the bone graft harvested from the fibula, the second image shows the graft before insertion into the sinus tarsi, and the third image shows the graft in its final position within the sinus tarsi.

The arthroereisis procedure consisted of the insertion of a titanium implant in sinus tarsi to limit excessive pronation and restore the medial longitudinal arch, as seen in Figure 2. Surgery was performed using a titanium, conical, threaded screw corresponding to a self-locking type of arthroereisis. Positioning was standard across surgeons, intraoperative fluroscopy was used the assess final position. Implant size was selected intraoperatively under fluoroscopic control to achieve optimal correction. Achilles tendon lengthening was performed selectively in patients with concomitant triceps surae tightness.

**Figure 3:**
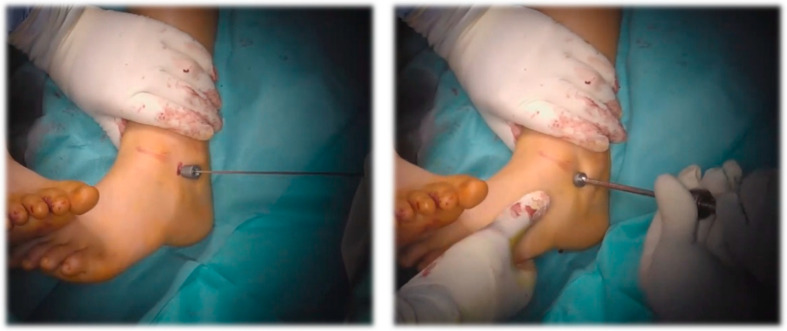
Intraoperative image showing the insertion of the screw over the guide wire immediately after tapping and determining the appropriate screw size, followed by threading it into the sinus tarsi.

Where possible, photographic evaluations were performed before and after surgery, as shown in Figure 3.

### 2.3. Radiographic Evaluation

Standardized weight-bearing anteroposterior and lateral radiographs of both feet were obtained preoperatively, at 3 and 6 weeks (standing AP and lateral), at 3 and 6 months, at 1 year, and annually thereafter.

**Figure 4:**
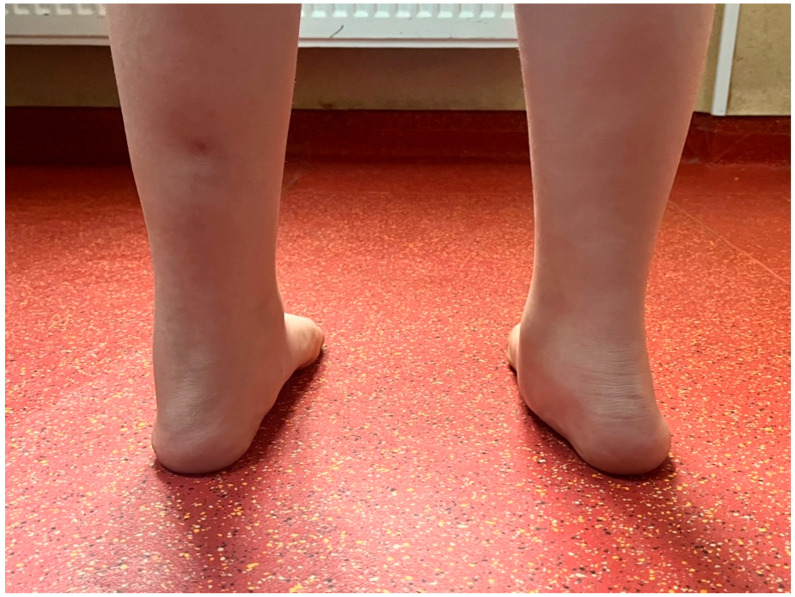
Preoperative image of an 8-year-old patient scheduled for right foot surgery, previously operated on the left side using the Grice procedure. The image shows proper hindfoot alignment and restoration of the medial longitudinal arch on the operated (**left**) side compared to the non-operated (**right**) side.

**Figure 5:**
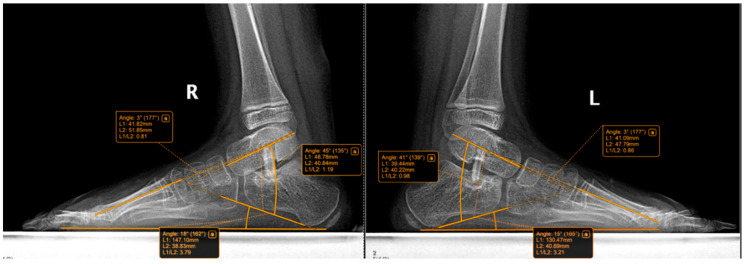
Radiographic image showing a patient who underwent bilateral Grice procedures. Using the digital system, manual measurements were performed for the pitch angle, Meary angle, and Kite’s angle. On the left foot, where the surgery was performed earlier, partial graft resorption can be observed. Postoperatively, Meary angles R: 3°, L: 3°; Kite angles to R: 45°, L: 41°; and calcaneal pitch to R: 18°, L: 15°. R = right; L = left.

**Figure 6:**
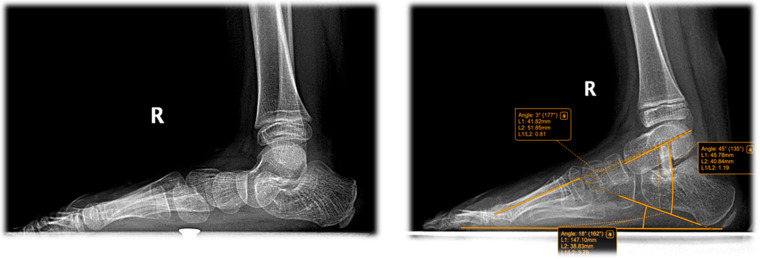
Radiographic images of a 7-year-old patient diagnosed with flat-valgus foot, operated on the right side using the Grice procedure. The (**left**) image shows the preoperative weight-bearing view, while the (**right**) image shows the postoperative result, demonstrating improved alignment and restoration of the normal foot arch. Postoperatively, Meary angle was 3°, Kite angle 45°, L: 45°; and calcaneal pitch 18°. R = right.

The following radiographic parameters were measured bilaterally using a digital radiological system, as seen in Figure 3, Figure 4, Figure 5 and Figure 6:

- Pitch angle (normal range 20–30°);
- Meary’s angle (normal <3°);
- Kite’s angle in frontal and profile projections (normal 25–40° and 35–50°, respectively);
- Talonavicular uncovering angle (normal <7°).

**Figure 7:**
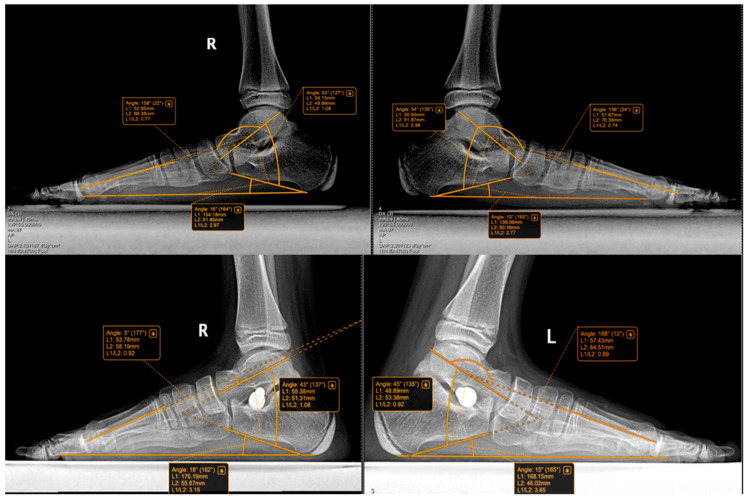
Bilateral weight-bearing lateral radiographs of an 11-year-old patient showing preoperative (**top row**) and postoperative (**bottom row**) images of a patient treated with the Arthroereisis procedure. Preoperatively, Meary angles were R: 22°, L: 24°; Kite angles R: 53°, L: 54°; and calcaneal pitch R: 16°, L: 15°. Postoperatively, Meary angles improved to R: 3°, L: 12°; Kite angles to R: 43°, L: 45°; and calcaneal pitch to R: 18°, L: 15°. R = right, L = left. Titanium implant can be observed in the Sinus tarsi.

**Figure 8:**
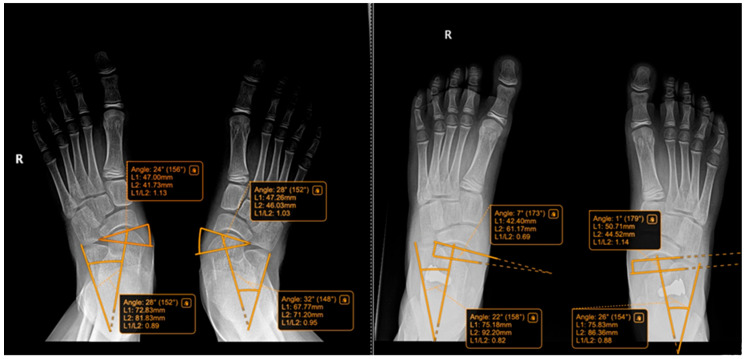
Radiographs of a 9-year-old patient treated with the Arthroereisis technique. The (**left**) image shows the preoperative view, and the (**right**) image shows the postoperative view, with measurements of the Kite angles (preoperative R: 28°, L: 32°; postoperative R: 22°, L: 26°) and talar uncovering angles (preoperative R: 24°, L: 28°; postoperative R: −7°, L: 1°). R = right.

In Figure 4 we present a comparative clinical view from behind, of the feet of an 8-year-old child who had undergone surgery for the left foot was awaiting surgery for the right foot. Some examples of measurements and comparative pre and post-operative are shown below in Figure 5, Figure 6, Figure 7 and Figure 8. 

All angles were measured in degrees (°). Measurements were performed independently by two pediatric orthopedic surgeons with more than five years of experience, and the mean of both readings was used for analysis. When both feet were operated, each side was analyzed independently.

### 2.4. Statistical Analysis

All statistical analyses were conducted using SPSS 26 Statistics (IBM Corp., Armonk, NY, USA) and verified in Python 3.13 (pandas, SciPy) for reproducibility. Continuous variables were tested for normality using the Shapiro–Wilk test. Descriptive results are presented as mean ± standard deviation (SD), 95% confidence intervals (CI), and range (min–max). No a priori sample size calculation was performed due to the retrospective design.

Within-group comparisons (preoperative vs. postoperative) were performed using the paired *t*-test. Between-group comparisons (Grice vs. Arthroereisis) of postoperative outcomes were assessed using the Welch *t*-test for unequal variances. A *p*-value < 0.05 was considered statistically significant.

### 2.5. Ethical Considerations

The study was conducted in accordance with the principles of the Declaration of Helsinki and approved by the Institutional Ethics Committee of Grigore Alexandrescu Children’s Hospital (approval code: 12/22.04.2024). All data were anonymized prior to analysis. Written informed consent for surgical treatment and the use of anonymized clinical and imaging data for research was obtained from the parents or legal guardians of all patients.

## 3. Results

Patient demographics and baseline characteristics are summarized in Table 1. Substantial differences between groups were observed at baseline. Patients undergoing the Grice procedure were younger and included all cases with cerebral palsy, whereas the arthroereisis group consisted exclusively of neurologically normal patients. Achilles tendon lengthening was performed in all Grice cases but in fewer than half of arthroereisis cases.

Radiographic outcomes demonstrated significant postoperative improvement within each surgical group across all measured parameters (Meary’s angle, calcaneal pitch, Kite’s angle, and talonavicular uncovering; all *p* < 0.05). These results are presented descriptively due to the marked baseline heterogeneity between groups.

No adjustment for confounding variables or clustering of bilateral feet was performed. Between-group comparisons should therefore be interpreted cautiously and are reported for exploratory purposes only.

Missing data were handled by complete case analysis. Only patients with available paired preoperative and postoperative radiographic measurements were included. No imputation of missing values was performed.

A total of 158 unique patients (were included in the analysis: 34 patients (54 feet) treated with the Grice procedure and 124 (232 feet) with arthroereisis.

The mean follow-up period for the Grice group was 9.0 years (95% CI, 7.53–10.47; range, 4–14 years), while for the arthroereisis group it was 7.2 years (95% CI, 6.71–7.61; range, 2–13 years).

Patient demographics and characteristics are presented in Table 1. The mean age at the time of surgery was 9.0 ± 3.1 years in the Grice group and 10.8 ± 2.6 years in the Arthroereisis group. The Grice cohort included 14 males and 20 females, while the Arthroereisis group comprised 84 males and 40 females. All patients with cerebral palsy (CP) were treated using the Grice technique (18 patients; 52.9%), whereas no Arthroereisis patients had CP. Achilles tendon lengthening was performed in all Grice cases (100%) and in 59 Arthroereisis cases. Regarding laterality, Grice procedures were more frequently bilateral (20/34), and Arthroereisis was also predominantly bilateral (108/124).

Significant postoperative improvements were observed in all evaluated radiographic parameters across both groups. In the Grice group, the mean Meary’s angle improved markedly, with an average correction of approximately 19.8° ± 9.2 on the right and 18.6° ± 8.7 on the left (both *p* < 0.001), as seen in Table 2.

The Arthroereisis group also demonstrated significant improvements, though of smaller magnitude, averaging 13.1° ± 7.5 on the right and 12.9° ± 7.2 on the left (*p* < 0.001). Between-group comparison showed a statistically significant difference in Meary’s angle correction favoring the Grice technique (*p* = 0.024) as seen in Table 3.

For the Pitch angle, both groups exhibited a postoperative increase consistent with improved longitudinal arch height. The mean increase was +9.2° ± 7.2 on the left and +8.7° ± 6.8 on the right in the Arthroereisis group, compared with +5.5° ± 6.2 and +5.3° ± 5.9 in the Grice group, respectively (*p* = 0.055 between groups).

Kite’s angle improved significantly in both frontal and profile planes, with no meaningful intergroup differences (*p* > 0.05). Similarly, reductions were noted in talonavicular uncovering and restoration of the Cyma line continuity across both cohorts, all achieving within-group significance (*p* < 0.001) but without statistical difference between procedures.

Overall, both surgical techniques produced significant postoperative radiographic correction of flatfoot parameters as seen in Table 4. The Grice arthrodesis resulted in greater angular realignment of the Meary axis, whereas Arthroereisis achieved higher Pitch angle recovery, particularly in older, neurologically normal patients. Despite these tendencies, no other between-group comparisons reached statistical significance, confirming that both approaches effectively corrected the deformity on radiographic grounds.

## 4. Discussion

This study demonstrates that both the Grice extra-articular subtalar arthrodesis and subtalar arthroereisis provide statistically significant radiographic correction in pediatric flexible flatfoot, confirming the effectiveness of both procedures in restoring normal foot alignment within their respective indication groups. The analysis of 158 unique patients revealed that each technique produced substantial improvements across all measured angular parameters, with distinct biomechanical advantages depending on patient age and deformity type.

In the Grice cohort, mean Meary’s angle improved from 20.4° (L) and 23.6° (R) preoperatively to 3.4° (L) and 3.8° (R) postoperatively (*p* < 0.001), corresponding to an average correction of approximately −19.8° ± 9.2 on the right and −18.6° ± 8.7 on the left. These values indicate nearly complete normalization (<3°), consistent with the structural realignment achieved by extra-articular subtalar grafting. Talonavicular uncovering also decreased markedly from 30.7° (L) and 26.1° (R) to 17.7° (L) and 18.4° (R) (*p* < 0.001), confirming medial column stabilization.

In the arthroereisis group, mean Meary’s angle decreased from 15.4° (L) and 16.1° (R) to 3.2° (L) and 3.1° (R) respectively (*p* < 0.001), with an average correction of −13.1° ± 7.5 (R) and −12.9° ± 7.2 (L). The Pitch angle increased significantly from 12.3° (L) and 12.1° (R) to 21.4° (L) and 21.2° (R) (*p* < 0.001) reflecting the restoration of longitudinal arch height. Kite’s angle improved in both planes (frontal: 40.6° → 30.4° L, 40.6° → 31.1° R; profile: 47.5° → 41.7° L, 48.4° → 41.5° R; all *p* < 0.001), confirming global correction of hindfoot valgus. These findings demonstrate that both operations achieved significant radiological normalization within their respective cohorts and indication groups.

Although all within-group changes were significant, inter-group comparisons identified specific tendencies. The Grice technique achieved greater correction of Meary’s angle (Δ = −19.8° vs. −13.1°, *p* = 0.024), indicating superior alignment of the talus-first metatarsal axis likely due to its rigid bony stabilization. Conversely, arthroereisis resulted in a larger Pitch angle increase (+9.2° vs. +5.5°, *p* = 0.055), reflecting enhanced restoration of the medial longitudinal arch without over-correction. Other angles (Kite, uncovering, Cyma line) improved comparably between procedures (*p* > 0.05), suggesting that both techniques achieve equivalent global correction of foot morphology within their respective indication groups.

Postoperative inter-group testing (Welch’s *t*-test) showed no significant differences in Meary’s, Pitch, or Kite frontal angles, but Grice exhibited higher Kite profile angles (46.2° L, 46.1° R) compared with arthroereisis (41.7° L, 41.5° R; *p* = 0.008 and *p* = 0.018), and higher talonavicular uncovering values (17.7° L, 18.4° R vs. 8.8° L, 9.0° R; *p* ≤ 0.001). These differences likely reflect the intrinsic rigidity of the Grice construct and the residual mobility preserved after arthroereisis.

The magnitude of correction observed aligns with published data. Mosca (2010) reported that calcaneal lengthening and Grice arthrodesis achieve durable angular normalization in valgus deformities, particularly in neuromuscular cases [1]. Bollmann et al. (2015) reviewed 92 Grice procedures and observed postoperative Meary’s angles of 2–5°, consistent with our mean 3.5° range [17].

For arthroereisis, Bernasconi et al. (2017) and Caravaggi et al. (2018) documented mean postoperative Meary’s angles of 2–4° and Pitch improvements of 7–10°, similar to our results [15,16]. Classical orthopedic references consistently define pediatric foot surgery as a balance between anatomical correction and preservation of growth potential, while differing slightly in their emphasis on surgical execution. McGlamry emphasizes meticulous anatomical dissection and biomechanical restoration as the foundation of durable correction in foot surgery [19], whereas Morrissy focuses on structured, step-by-step operative planning and detailed visual guidance adapted to pediatric anatomy [20]. In contrast, Jianu highlights pragmatic, experience-based adaptations of these principles within pediatric orthopedic practice, with particular attention to surgical timing and technical simplicity [21]. Despite these nuanced differences, all three sources converge on the importance of precise alignment, careful soft-tissue handling, and age-appropriate techniques as key determinants of successful outcomes in pediatric foot surgery. Recent systematic reviews confirm arthroereisis as an effective, minimally invasive option providing reproducible radiographic correction with low complication rates [22,23]. The slightly larger residual Meary and talonavicular angles in our arthroereisis group may reflect inclusion of older, idiopathic cases with partially adaptive soft-tissue structures.

The data support the concept that both operations can achieve physiological alignment but should be applied selectively. Grice arthrodesis remains particularly suited for younger or neurologically impaired patients requiring permanent stabilization, while arthroereisis provides adequate correction for flexible deformities with preserved subtalar motion. The statistically greater Meary correction after Grice corroborates its effectiveness in more severe deformities, whereas the higher postoperative Pitch following arthroereisis indicates better arch reconstitution in flexible cases.

No major between-group differences in final radiological alignment confirm that both methods reach equivalent corrective goals when appropriately indicated.

These patterns mirror prior biomechanical and radiographic analyses. Mosca (2010) [1] emphasized that the Grice extra-articular arthrodesis remains the most reliable option for stabilizing valgus deformities in patients with neuromuscular or rigid flatfoot, providing long-term structural correction without compromising ankle motion. Bollmann et al. (2015) [17] In their series, the lateral talo–first metatarsal angle improved from a mean of −36.98° to −12.32°, the calcaneal pitch increased from 2.95° to 7.55°, and the lateral talo–calcaneal angle decreased from 49.52° to 31.49°, reflecting substantial correction of hindfoot valgus and restoration of the medial longitudinal arch. Comparable trends were documented in our study, where the Meary angle improved from 20–23° preoperatively to 3–4° postoperatively, and the calcaneal pitch rose from 12–13° to 18–19°, indicating near-physiological sagittal alignment of the medial column. Furthermore, the reductions in the anteroposterior and lateral Kite angles and in talonavicular uncovering mirrored the radiographic improvements described by Bollmann and colleagues. Taken together, these findings underscore the reproducibility and efficacy of the Grice extra-articular arthrodesis in achieving durable realignment of valgus deformities of the hindfoot in the pediatric population.

For arthroereisis, our findings correspond to modern literature emphasizing minimally invasive correction. Bernasconi et al. (2017) [15] who highlighted the role of subtalar arthroereisis as a minimally invasive and reversible technique that achieves reliable correction of flexible flatfoot in pediatric and adult populations. They reported significant postoperative improvement in both radiographic and clinical outcomes, with correction of Meary’s and talonavicular angles comparable to more extensive surgical procedures. In our cohort, similar trends were observed, as postoperative angular measurements approached physiological ranges with low complication rates and stable correction over time. These parallels reinforce the growing evidence that arthroereisis can provide durable and functionally satisfactory results when appropriately indicated, particularly in flexible deformities without severe structural or neurological involvement.

Caravaggi et al. (2018) [16] emphasized the dynamic nature of subtalar arthroereisis, which corrects deformity through a temporary, implant-based modulation of motion rather than rigid fixation. This dynamic correction permits progressive realignment during growth while maintaining subtalar joint mobility and allowing bone remodeling. Such characteristics make the procedure particularly suited for pediatric patients, as also observed in our cohort, where postoperative alignment was achieved without the loss of joint function, which may explain the higher postoperative Pitch values and improved arch congruency observed in our patients. Smith et al. (2021) [22], in a systematic review encompassing over 1200 cases, reported mean improvements of 9° in Meary’s angle and 3.5° in Pitch. The physiological correction and joint preservation seen in our cohort are thus in line with the recognized advantages of the arthroereisis technique.

**Figure 9:**
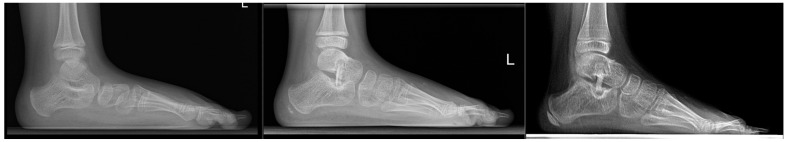
Female patient diagnosed at age 5 (first image), operated at age 6 (second image), with follow-up at age 7 showing partial resorption of the fibular graft. Long-term outcome is favorable, with maintenance of the achieved correction. L = left.

When comparing the two techniques, our data echo the findings of Giannini and Caravaggi (2018) [16], who proposed that the choice between procedures should be tailored to the underlying etiology and rigidity of deformity. Grice arthrodesis achieves lasting correction but sacrifices subtalar mobility, making it suitable for neuromuscular or rigid cases, whereas arthroereisis offers dynamic realignment ideal for idiopathic flexible flatfoot. The small but consistent differences between postoperative angular outcomes highlight this complementarity rather than competition between techniques. Eventually, with the Grice procedure, the fibular graft will undergo resorption; however, the correction will be maintained, as shown in Figure 9.

Our results are consistent with the findings of a previous study by our team, which reported that subtalar arthroereisis in children and adolescents with flexible flatfoot led not only to radiographic correction but also to significant improvements in sports performance, foot aesthetics, and overall quality of life [24]. Similarly to our findings, the present study confirms that restoring the physiological alignment of the foot through minimally invasive stabilization translates into both functional and psychosocial benefits. These aspects are particularly relevant in the pediatric population, where rapid recovery and early return to normal activities play an essential role in long-term satisfaction and treatment success.

Although both procedures aim to correct symptomatic flexible flatfoot, the indications for Grice arthrodesis and arthroereisis differed substantially in our population and reflect established biomechanical and clinical principles. In our series, Grice arthrodesis was selected primarily for younger patients and those with neuromuscular impairment, particularly cerebral palsy, where valgus deformity tends to be more rigid, progressive, and associated with abnormal muscle tone. These cases require a stable extra-articular construct capable of maintaining correction during growth, which explains why all CP patients in our cohort underwent the Grice technique. Conversely, arthroereisis was used predominantly in older children with flexible idiopathic deformities, where subtalar motion is preserved and a dynamic, minimally invasive correction is preferable. The larger postoperative Pitch angle observed in this group supports the notion that arthroereisis is most effective in patients with flexible arch collapse but intact neuromuscular control. Therefore, our findings reinforce the age- and etiology-based indications proposed in previous literature, while providing objective radiographic confirmation that the two procedures address distinct clinical subsets rather than interchangeable surgical options.

**Figure 10:**
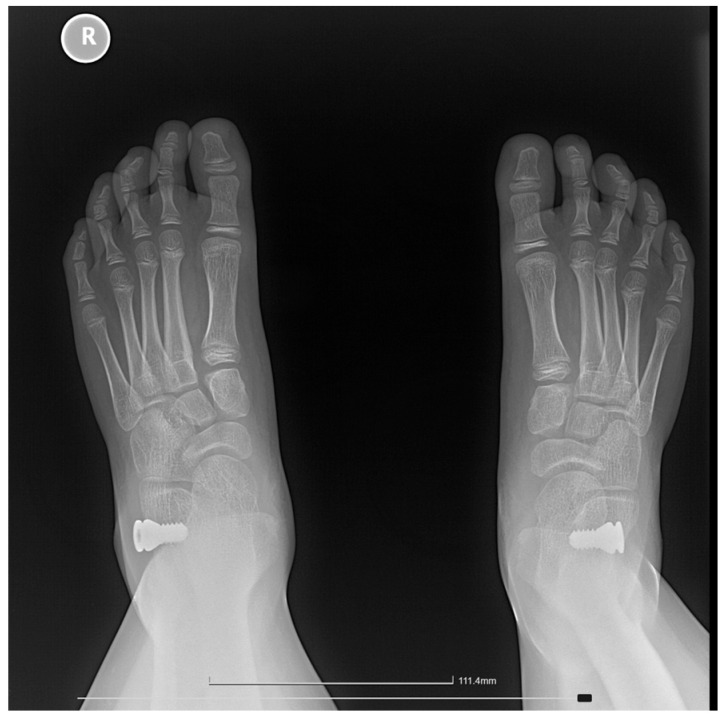
Implant migration observed on the right side after the Arthroereisis procedure, occurring within the first three weeks after surgery. R = right.

Postoperative complications were infrequent across both groups. One patient in the arthroereisis group experienced implant migration as seen in Figure 10, which required revision surgery. Another case of superficial wound infection occurred in the Grice group and resolved with targeted antibiotic therapy and local care. No cases of neurovascular injury, persistent pain, or recurrence were observed during the follow-up period. Overall, both techniques demonstrated a favorable safety profile within their respective indication groups.

Importantly, direct comparison between the two surgical techniques is limited by confounding by indication. The Grice procedure was preferentially used in younger patients and those with neuromuscular impairment, whereas arthroereisis was reserved for older children with idiopathic flexible flatfoot. These populations are inherently non-comparable, and the observed differences in radiographic correction may reflect baseline pathology rather than true procedural superiority.

All patients were monitored from the time of surgery until reaching 18 years of age. The shorter follow-up in the arthroereisis cohort reflects the more recent implementation of this minimally invasive technique in our department, compared with the longer-established Grice procedure.

This study has several important limitations. First, its retrospective observational design precludes causal inference. Second, the two treatment groups were inherently non-comparable due to confounding by indication, particularly the inclusion of all cerebral palsy cases in the Grice group and exclusively idiopathic cases in the arthroereisis group. Third, no adjustment was performed for baseline differences, clustering of bilateral feet within patients, or potential surgeon-related effects, limiting the validity of between-group comparisons.

Fourth, outcomes were limited to radiographic parameters, and no standardized clinical or patient-reported outcome measures were available. As radiographic correction does not necessarily translate into functional improvement or patient satisfaction, the clinical relevance of the findings should be interpreted with caution. Finally, missing data were addressed using complete-case analysis, which may introduce selection bias and limit generalizability. No multivariable adjustment was performed due to confounding by indication and baseline non-comparability between treatment groups, and all between-group analyses should be interpreted as descriptive.

The retrospective design, moderate sample disparity between groups (34 vs. 124 patients), and reliance on radiographic rather than functional outcomes. Follow-up duration varied, and implant type was not stratified in the arthroereisis cohort. Nevertheless, the dataset’s size and bilateral measurement approach strengthen the reliability of statistical findings. A major limitation of this study is the absence of standardized pain or functional outcome measures such as the AOFAS Hindfoot Scale, the Oxford Ankle Foot Questionnaire for Children (OxAFQ-C), or visual analogue pain scores. Although radiographic correction is essential for understanding biomechanical improvement, it does not fully capture the patient’ s functional recovery or subjective satisfaction. Previous studies have demonstrated that both Grice arthrodesis and arthroereisis lead to significant improvements in pain, activity tolerance, and quality of life [24]; however, the retrospective nature of our cohort and incomplete documentation of PROMs prevented their inclusion in the present analysis. Future prospective studies will incorporate systematic functional scoring to provide a more comprehensive assessment of clinical outcomes alongside radiographic parameters.

Both the Grice extraarticular subtalar arthrodesis and subtalar arthroereisis procedures achieved significant radiographic correction of symptomatic flexible flatfoot in children within their respective indication groups. Across all measured parameters Pitch, Meary, Kite, and talonavicular uncovering angles postoperative values approached normal reference ranges, reflecting restoration of the medial longitudinal arch and hindfoot alignment.

The Grice technique demonstrated reliable long-term outcomes with consistent correction in both sagittal and coronal planes, particularly in patients with neuromuscular or structural etiologies. Arthroereisis, a minimally invasive alternative, provided comparable alignment improvement with shorter operative times, reduced surgical morbidity, and good maintenance of correction during growth.

Despite the smaller cohort and longer follow-up in the Grice group, both interventions proved effective and safe in pediatric patients. The few complications observed one implant migration and one superficial wound infection were manageable without compromising final results.

Continued prospective evaluation with functional and long-term radiological follow-up is warranted to refine patient selection and confirm durability of correction into skeletal maturity.

## 5. Conclusions

In this retrospective observational study, both Grice extra-articular subtalar arthrodesis and subtalar arthroereisis were associated with significant radiographic improvement within their respective patient populations. Due to substantial baseline differences and confounding by indication, no causal or comparative effectiveness conclusions can be drawn. The results should be interpreted as descriptive and hypothesis-generating. Future prospective studies incorporating standardized clinical outcomes and appropriate statistical adjustment are required to determine the relative effectiveness of these procedures. Each surgical procedure has its own advantages and limitations, and optimal treatment planning should be tailored to the individual patient, considering clinical presentation, etiology of the deformity, age, and functional demands.

## Figures and Tables

**Table 1:** Patient demographics and group characteristics are shown, including mean age at surgery, sex distribution, laterality, neurological status (CP), and the presence of Achilles tendon lengthening.

Procedure	Arthroereisis	Grice
Age (mean)	10.84	9
Age (SD)	±2.55	±3.08
Male	84	20
Female	40	14
CP	0	18
Achilles tendon lengthening	59	34
Bilateral	108	20
Unilateral	16	14
Total patients	124	34

**Table 2:** Preoperative and postoperative measurements for the Grice procedure, including calcaneal pitch, Meary angle, Kite angle (AP and lateral), talonavicular uncovering, and the side on which the measurements were taken (left or right).

GRICE Angle Type	Preoperative			Postoperative		
	Mean (°)	95% CI	(Min–Max)	Mean (°)	95% CI	(Min–Max)
Pitch left (normal 20–30°)	13.65°	[11.78–15.52]	(5–24°)	19.12°	[16.76–21.48]	(8–23°)
Pitch right (normal 20–30°)	12.12°	[10.59–13.65]	(6–20°)	18.44°	[15.59–21.28]	(3–22°)
Meary left (normal <3°)	20.41°	[16.60–24.22]	(7–40°)	3.35°	[2.28–4.43]	(2–10°)
Meary right (normal <3°)	23.59°	[19.22–27.96]	(4–41°)	3.75°	[2.48–5.02]	(1–10°)
Kite AP left	35.94°	[33.93–37.94]	(29–46°)	29.20°	[25.21–33.19]	(12–35°)
Kite AP right	37.88°	[33.57–42.18]	(23–45°)	33.62°	[29.17–38.08]	(14–40°)
Kite L left	52.41°	[48.77–56.06]	(35–70°)	46.24°	[43.38–49.10]	(33–51°)
Kite L right	52.65°	[49.24–56.06]	(37–67°)	46.06°	[42.74–49.39]	(30–51°)
Talonavicular uncovering left	30.69°	[27.49–33.88]	(17–46°)	17.67°	[14.51–20.82]	(3–26°)
Talonavicular uncovering right	26.06°	[20.39–31.74]	(13–62°)	18.44°	[13.94–22.94]	(1–25°)

**Table 3:** Preoperative and postoperative measurements for the Arthroereisis procedure, including calcaneal pitch, Meary angle, Kite angle (AP and lateral), talonavicular uncovering, and the side on which the measurements were taken (left or right).

Arthroeresis	Preoperative			Postoperative		
Angle Type	Mean (°)	95% CI	(Min–Max)	Mean (°)	95% CI	(Min–Max)
Pitch left (normal 20–30°)	12.30°	[11.38–13.22]	(3–28°)	21.38°	[20.38–22.39]	(3–30°)
Pitch right (normal 20–30°)	12.09°	[11.14–13.03]	(2–30°)	21.24°	[20.25–22.23]	(5–30°)
Meary left (normal <3°)	15.36°	[14.04–16.68]	(3–50°)	3.23°	[2.81–3.64]	(0–9°)
Meary right (normal <3°)	16.15°	[14.83–17.46]	(1–51°)	3.13°	[2.73–3.53]	(0–11°)
Kite F left	40.66°	[39.56–41.76]	(25–57°)	30.44°	[29.79–31.08]	(21–40°)
Kite F right	40.55°	[39.36–41.74]	(26–53°)	31.14°	[30.28–31.99]	(15–44°)
Kite P left	47.50°	[46.31–48.70]	(34–67°)	41.68°	[40.77–42.60]	(27–55°)
Kite P right	48.40°	[47.31–49.49]	(36–70°)	41.48°	[40.59–42.37]	(29–57°)
Talonavicular uncovering left	19.46°	[17.80–21.13]	(5–59°)	8.76°	[7.73–9.78]	(0–27°)
Talonavicular uncovering right	17.52°	[16.00–19.04]	(0–56°)	9.00°	[7.85–10.15]	(0–31°)

**Table 4:** Postoperative radiographic angle measurements in the Grice and Arthroereisis groups, including the number of cases (N), mean values, and *p*-values for comparison. The evaluated parameters were: calcaneal pitch (left and right), Meary angle (left and right), Kite angle (AP and lateral, left and right), and talonavicular uncovering (left and right).

Angle (Postop)	N (Grice)	Mean (Grice)	N (Arthroereisis)	Mean (Arthroereisis)	*p*
Pitch left (postop)	34	19.11°	120	21.38°	0.0969
Pitch right (postop)	32	18.43°	119	21.24°	0.0838
Meary left (postop)	34	3.35°	120	3.22°	0.8300
Meary right (postop)	32	3.75°	119	3.12°	0.3709
Kite AP left (postop)	30	29.2°	119	30.43°	0.5582
Kite AP right (postop)	32	33.62°	118	31.13°	0.2979
Kite Lateral left (postop)	34	46.23°	120	41.68°	0.0077
Kite Lateral right (postop)	32	46.06°	119	41.47°	0.0182
Talonavicular uncovering left (postop)	30	17.66°	119	8.75°	0.0001
Talonavicular uncovering right (postop)	32	18.43°	118	9°	0.0010

## Data Availability

Data are available upon request.
